# Large scale simulation of labeled intraoperative scenes in unity

**DOI:** 10.1007/s11548-022-02598-z

**Published:** 2022-03-30

**Authors:** Thomas Dowrick, Brian Davidson, Kurinchi Gurusamy, Matthew J Clarkson

**Affiliations:** 1 Wellcome EPSRC Centre for Interventional and Surgical Sciences, UCL, London, UK; 2 Division of Surgery and Interventional Science, UCL, London, UK

**Keywords:** Simulation, IGS, Liver surgery

## Abstract

**Purpose:**

The use of synthetic or simulated data has the potential to greatly improve the availability and volume of training data for image guided surgery and other medical applications, where access to real-life training data is limited.

**Methods:**

By using the Unity game engine, complex intraoperative scenes can be simulated. The Unity Perception package allows for randomisation of paremeters within the scene, and automatic labelling, to make simulating large data sets a trivial operation. In this work, the approach has been prototyped for liver segmentation from laparoscopic video images. 50,000 simulated images were used to train a U-Net, without the need for any manual labelling. The use of simulated data was compared against a model trained with 950 manually labelled laparoscopic images.

**Results:**

When evaluated on data from 10 separate patients, synthetic data outperformed real data in 4 out of 10 cases. Average DICE scores across the 10 cases were 0.59 (synthetic data), 0.64 (real data) and 0.75 (both synthetic and real data).

**Conclusion:**

Synthetic data generated using this method is able to make valid inferences on real data, with average performance slightly below models trained on real data. The use of the simulated data for pre-training boosts model performance, when compared with training on real data only.

## Introduction

For researchers working on machine learning/AI applications in Image Guided Surgery (IGS), the lack of application specific training data is a perennial issue. In addition, the time-consuming process of manually labelling data is especially challenging for medical data, as labelling complex intraoperative scenes or radiological data typically requires the expertise of a trained clinician.

The use of synthetic data for model training has benefited the wider computer vision community [1, 2], but there have been limited applications in the IGS field. The application of image to image transfer, and video style transfer [3, 4] are a promising approach to generate fully labelled data, but large amounts of data are required to train these networks initially, which limits their use to applications where training data is already available, and it is difficult to introduce variance into the data that is not present in the training data.

An alternative approach, described here, is to generate synthetic data, with little or no input form existing clinical data sets. The approach makes use of the Unity (https://unity.com/) games engine, and in particular the Unity Perception package (https://github.com/Unity-Technologies/com.unity.perception). Perception provides a framework for generating large datasets, by randomising parameters (model size/shape/position, textures, lighting etc.) and automatic labelling of the scene. Combined with Unity’s state of the art functionality for rendering, lighting, particle systems and animation this provides an excellent platform for researchers to rapidly generate large datasets for model development.

In this work, an initial application of this approach to the problem of liver segmentation from laparoscopic video images is considered. This was chosen due to the availability of a well-labelled dataset of real surgical images for comparative purposes, but the approach could easily be extended to other organs and labelling scenarios.

**Fig. 1:**
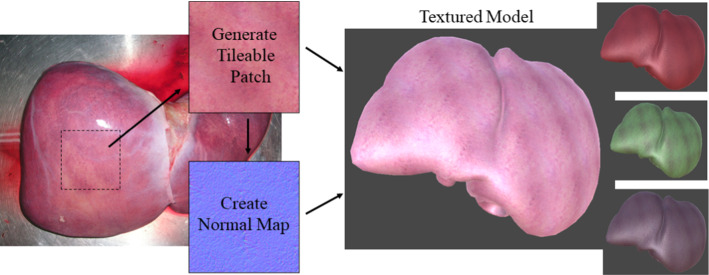
Texture generation process. A sample patch is cropped from a relevant image (Source: Wikipedia), converted to a tileable pattern and a normal map generated. Texture variety is increased further by randomisation of RGB parameters at simulation time

**Fig. 2:**
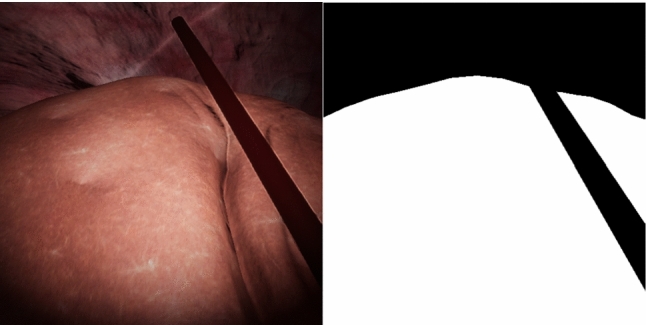
Sample synthetic data and liver segmentation

**Fig. 3:**
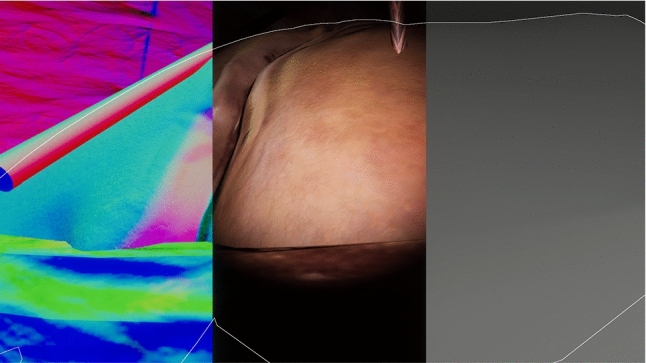
Normal, depth and contour labels can be generated in Unity

**Table 1:** Mean DICE score per patient, for the three training scenarios

	P11	P12	P13	P14	P15	P16	P17	P18	P19	P20	Mean
Syn	0.74	0.75	0.70	0.56	**0.83**	**0.71**	0.32	0.37	**0.32**	**0.57**	0.59
Real	**0.85**	**0.91**	**0.91**	**0.68**	0.68	0.48	**0.51**	**0.64**	0.22	0.53	**0.64**
Both	0.87	0.92	0.92	0.78	0.88	0.79	0.56	0.69	0.38	0.75	0.75

Bold indicates the highest DICE score, between Synthetic and Real data

## Method

A Unity scene was created to mimic a simplified intraoperative view of the liver, comprising a liver model, layer of fat surrounding the liver, a laparascopic tool and a Unity spot light to represent the scope light source, all enclosed within a torso shaped background surface.

To demonstrate that suitable data can be generated without requiring application specific training data to act as a starting point, all models and textures used for simulation were acquired from online stores/repositores.

A liver model, complete with 8k textures was purchased from the TurboSquid model store (https://www.turbosquid.com). All other models were created using 3D primitives in Unity/Blender.

Additional textures for the liver, fat and background were either sourced from existing texture libraries (https://www.texturecan.com/) or synthesised from relevant images to create a tileable pattern (Fig. 1). Where normal maps were not available for textures, these were generated using CrazyBump.

The following parameters were randomly varied during simulation—liver texture and normal map; tiling and RGB offset of liver texture; background texture and normal map; position of laparoscopic tool; intensity (500–1500), range (0.75–1.25) and outer angle (50–90) of spot light; camera position and orientation within the torso, distortion (0–0.65) and motion blur (0–0.5). Apart from the textures/normal maps, which were taken from a discrete set, all variables were linearly sampled from a continuous distribution. Quoted figures give the range of values used for the respective Unity parameters. Relevant code is available at https://github.com/UCL/Synthetic_Liver_IPCAI_2022.

### Model training

A dataset of 1835 images of laparascopic liver surgery in 20 patients were manually segmented to identify the liver. This was split into training (10 patients, 950 images) and evaluation (10 patients, 885 images) sets. Fifty thousand synthetic images were generated in Unity (time required for generation time was in the order of 10 min), with a corresponding segmentation image in each case (Fig. 2) Additional examples can be viewed online (https://youtu.be/JVsyZRoHxz8), and the full dataset is available at weiss-develop.cs.ucl.ac.uk/liver-ipcai-2022/synthetic-liver-images.zip. For demonstration purposes, labels were generated for the liver contour, depth map and normal map (Fig. 3). Three different training scenarios were run—(1) Train on the 50,000 synthetic images only. (2) Train on the 950 surgical images only. (3) Pre-train on synthetic data, post-train on real surgical images.

In each case, the network was evaluated using the 10 patients in the evaluation set, using a U-Net (https://github.com/milesial/Pytorch-UNet) over 10 epochs, learning rate of 0.001, batch size of 10, with RMSProp optimizer.

## Results

DICE scores were calculated per patient in the validation set, for the three approaches (Table 1). Training with both datasets always produces better results, but when comparing synthetic only and real only training, the best approach varied, with synthetic data performing best in 4 out of 10 cases.

## Discussion

The presented work demonstrates that the generated synthetic data can be used in its own right to make valid predictions, outperforming real data in some cases, and that it can also be used to pre-train a model for increased performance. Ongoing work in this area is looking into modelling additional organs, evaluating performance with different deep learning algorithms, evaluating trained models in clinical scenarios, and into incorporating Unity functionality to render particles (e.g. blood) and soft body physics effects for added realism.
